# 

**Published:** 1910-10-29

**Authors:** 


### CONTENTS.

| | PAGE |
|---|---|
| **The Crippen Case** | 121 |
| **Medical Tuition by Correspondence** | 122 |
| **Annotations—** | |
| Man, Woman, and Athletics—Train Lavatories—The Local Government Board Report | 123 |
| **Medical Opinion and Movement** | 124 |
| **Hospital Clinics—** | |
| Mental Evolution and the Insanities of To-day. By Robert Jones, M.D., F.R.C.P., F.R.C.S. | 127 |
| **Practical Notes on Diagnosis and Treatment** | 130 |
| **Medicine—** | |
| Multiple Crises in Tabes Dorsalis | 131 |
| Melanuria | 131 |
| **Laryngology and Rhinology—** | |
| Difficulties in Removing the Tracheotomy Tube after Diphtheria | 132 |
| **Pathology—** | |
| Hæmatology—IX. | 13 |
| **Toxicology—** | |
| Severe Poisoning by Homatropin used as an Eye Instillation | 134 |
| **News and Events of the Week** | 135 |
| **Obituary** | 136 |
| **The Week's Work—** | |
| The late Prince Francis of Teck—Hospital Abuse and the Minority Report—The Minority Report and the Conference—A Bad System—The Newcastle Infirmary Chapel—The Necessity for Toleration—This Week's Drug Market | 137 |
| **Special Institutional Article—** | |
| The Coming of a Unified County Medical Service, and How it will Affect the Voluntary Hospital | 139 |
| **Editor's Letter-Box—** | |
| The Danger of Flannelette | 145 |
| **Royal Albert Edward Infirmary, Wigan, New Mortuary** | 145 |
| **Gifts and Bequests** | 146 |
| **Jottings** | 146 |
| **The Hospital World** | 147 |
| **Parliament and Publications—** | |
| The Hospitals of Kedah | 148 |
| **Public Grants for Voluntary Hospitals** | 149 |
| **North Midland Poor-Law Conference** | 150 |
| **New Appliances and Things Medical** | 150 |
| **Institutional Notes and News** | 151 |

